# Alternative Clinker Technologies for Reducing Carbon Emissions in Cement Industry: A Critical Review

**DOI:** 10.3390/ma15010209

**Published:** 2021-12-28

**Authors:** Mónica Antunes, Rodrigo Lino Santos, João Pereira, Paulo Rocha, Ricardo Bayão Horta, Rogério Colaço

**Affiliations:** 1 IDMEC, Instituto Superior Técnico, Universidade de Lisboa, Av. Rovisco Pais, 1049-001 Lisboa, Portugal; monica.h.antunes@tecnico.ulisboa.pt; 2 CIMPOR Portugal Holdings, SGPS S.A., Avenida José Malhoa, 22, 1099-020 Lisboa, Portugal; RLSantos@cimpor.com (R.L.S.); jpereira@cimpor.com (J.P.); procha@cimpor.com (P.R.); 3 Instituto Superior Técnico, University of Lisbon, Av. Rovisco Pais, 1049-001 Lisboa, Portugal; Teresa.Empis@Cires.pt

**Keywords:** alternative clinkers, CO_2_ emissions, OPC, process electrification

## Abstract

Currently, the production of one ton of ordinary Portland cement (OPC) releases considerable amounts of CO_2_ into the atmosphere. As the need and demand for this material grows exponentially, it has become a challenge to increase its production at a time when climate-related problems represent a major global concern. The two main CO_2_ contributors in this process are fossil fuel combustion to heat the rotary kiln and the chemical reaction associated with the calcination process, in the production of the clinker, the main component of OPC. The current paper presents a critical review of the existent alternative clinker technologies (ACTs) that are under an investigation trial phase or under restricted use for niche applications and that lead to reduced emissions of CO_2_. Also, the possibility of transition of clinker production from traditional rotary kilns based on fuel combustion processes to electrification is discussed, since this may lead to the partial or even complete elimination of the CO_2_ combustion-related emissions, arising from the heating of the clinker kiln.

## 1. Introduction

The history of cement has had a major impact on the progress of our civilization during the last century [1]. This cheap mineral binder, when in contact with water, goes through a set of relatively complex physicochemical reactions, that result in a stone-like hard material. This allows the production of mortars, cement mixed with water and sand, and concrete, cement mixed with water, sand and aggregates such as gravel and slag [2]. Concrete is, not only extremely resilient and durable but can also bear heavy compressive loads and resist severe environmental conditions. These set of properties combined allowed concrete to position as the man-made most widely used material in the world [3]. Due to the high demand for concrete, 4.3 Gigatons of cement were estimated to be produced globally during the year 2020 [4].

However, the amount of CO_2_ released during the production of OPC has a very strong environmental impact. In fact, the production of one ton of clinker releases about 0.83 tons of CO_2_ and the production of one ton of OPC releases about 0.54 ton of CO_2_ [5] making this industry responsible for 5% to 8% of total anthropogenic greenhouse gases [6] The two main sources of CO_2_ emissions resulting from cement production are: (i) the decarbonation of limestone since CaCO_3_ is decomposed into CaO and CO_2_ at temperatures above 550 °C, with this contribution representing about 60 to 65% of the total CO_2_ emissions [7] and (ii) the fossil fuel combustion to heat the cement kiln, which is responsible for the remaining 35 to 40% of the emissions.

Hence, within the scope of the 2030 United Nations (UN) agenda [8] and also driven by the increasingly higher CO_2_ emission taxes it has become a target and a challenge for the cement industry to develop new binders with a lower ecologic footprint, that can be produced at a large scale, so that it can be used as a commodity, without compromising the technical, economic and workability qualities characteristic of OPC. As it became clear from the 26th UN Climate Change Conference of the Parties (COP26), held in Glasgow in November 2021, managing the pressure for the unavoidable need of social development together with the agendas of environmental sustainability and climate change control will be the challenge of the century, and the cement industry itself is a relevant key player in this fundamental discussion.

Until now, the methods that have been studied to mitigate CO_2_ emissions in cement production follow five main approaches [9]:

- Reduction of the cement-to-clinker ratio, by replacing clinker with supplementary cementitious materials (SCM’s);
- The use of alternative fuels in the production of clinker together with the increase of energy efficiency of the kiln process;
- Development of alternative clinker technologies (ACTs), that lead to lower CO_2_ emissions.
- Carbon capture, use and storage (CCUS), i.e., the sequestration and use of the emitted CO_2_ for specific applications;
- Electrification of the clinker production process, especially if renewable electricity produced from non-fossil energy sources is used;

CCUS technologies consist of the capture, transport, use and storage of CO_2_ [10]. More interestingly, a number of research projects have also explored the CCS possibility, bringing insights into the use of CO_2_ for other applications, namely the production of hydrocarbons or alcohols, through the reaction of CO_2_ with H_2_ [11]. Yet, in general, CCUS technologies are still costly and in a demonstration stage of Technology Readiness Level (TRL) [12]. On the other hand, the cement kiln main stack flue gases have high amounts of impurities such as N_2_, SOx, NOx and CO [13], which make difficult the capture and direct use of pure CO_2_. Further research and development will therefore be needed to reduce the cost of the capture step and to increase the TRL of CCUS technologies to render them an economically viable solution to be applied in the cement industry. The work of Plaza et al. [10] provides a comprehensive and interesting overview of the CCUS technologies that have been evaluated in the cement industry at the pilot scale and also the plans for further industrial implementation.

Regarding SCMs, it is worth mentioning the main conclusions of the working group initiated by the United Nations Environment Program-Sustainable Building and Climate Initiative (UNEP-SBCI) published in 2018 [14]. The group concluded that Portland-based cement approaches will dominate in the near future due to economies of scale, levels of process optimization, availability of raw materials and market confidence, but that two product-based approaches can deliver substantial additional reductions in their global CO_2_ emissions, over the next 20–30 years: increasing the use of low-CO_2_ additives, or supplementary cementitious materials (SCMs), as partial replacements for Portland cement clinker and more efficient use of OPC clinker. Several types of SCMs may be added to cement during concrete production, including: rice husk ash, silica fume, fly ash and ground granulated blast furnace slag (GGBFS) [15]. For rice husk, the recommended replacement level is 30%, this SCM densifies the concrete matrix and reduces the volume of voids [16]. For silica fume, previous results showed that a replacement of 5% produces the best performance, with an increase of workability, strength and durability of the material [17]. Fly ash may replace up to 70% of the cement, allowing the production of so-called high-volume fly ash concrete (HVFA) [18]. Finally, GGBFS can replace up to 40% of cement without the need for a superplasticizer [19]. However, a major disadvantage of SCMs, pointed out by the (UNEP-SBCI) [14] working group, is that high blended cements may exhibit slow early-age strength development and uncertainty on the long-term durability [20]. Also, the concrete produced with OPC+SCM may present lower flowability [12], hampering the pumping, spreading, molding and compaction of the material [20]

The mix of fossil fuels commonly used to heat the cement kiln (e.g., petcoke, coal, natural gas, fuel oil, mazut, etc.) are already being replaced, to some extent, with alternative fuel such as waste tires, sewage sludge, animal meal, waste oil, waste paper, plastics, textiles, agriculture and biomass wastes, amongst other. Nevertheless, these alternative fuel solutions only represent up to 10% of the reduction of the total CO_2_ emissions in cement production [21]. According to CEMBUREAU’s Roadmap to Carbon Neutrality in 2050, altogether, the replacement of fossil fuels by non-recyclable and biomass waste, and the use of alternative raw materials, will deliver 15% of the CO_2_ emissions reduction in the cement industry in 2050 [22].

A much more interesting approach in what concerns energy supply is the electrification of the process, especially if the electricity comes from low-carbon or non-fossil sources [23]. We will come to the topic of electrification at the end of this paper due to the key importance that it may represent in the near future, particularly having in consideration the European Green Deal agenda and the ongoing green agendas in the rest of the world. Nevertheless, we must have taken into consideration two fundamental aspects regarding the electrification of the cement industry:

- The best available technology (BAT) of OPC production cannot be easily converted into a fully electrified process, since it would require very significant changes not only in the technology itself but also in the dimensioning of the industrial installation due to the lower volume of material (gases and dust) in circulation.
- At least 60% of the released CO_2_ comes from the decarbonation stage, while only 35 to 40% of the remaining CO_2_ emissions come from the energy needed to promote the high-temperature reactions that characterize the clinkering stage. Thus, acting in the material composition stage is mandatory to achieve a significant reduction of CO_2_ emissions in cement production. Therefore, at the present point, other emerging technologies or alternative materials will also play an important role in emissions mitigation in the medium/longer term.

This paper intends to provide a critical review on this last topic: the use of alternative binders, focusing on the threats and opportunities that can be open in a long-term vision, at a moment in which the cost of CO_2_/ton almost reaches the production cost of one ton of clinker.

Concerning ACTs, these approaches have to be able to generate, not only a similar economic value to that of OPC, accomplishing the necessary reduction of CO_2_ emissions, but at least, present a similar competitive performance. Some of the most critical properties, which must be taken into consideration are:

- Mechanical performance, both at early ages and later ages;
- Rheological performance, enabling an adequate pumping, spreading, molding and compaction of the material [20];
- Reduced chemical shrinkage, since this phenomenon causes serious loss on the durability and bearing capacity of concrete structure, increasing the risk for cracking and decreasing the lifespan of the structures [24],
- Chemical stability, which will be translated into structure durability when submitted to the natural elements.

The fulfillment of the before mentioned requirements is the basis for the development of new alternative clinker technologies, however, any emerging technological solution should be able to compensate for the substitution of the existing OPC production BAT in terms of replacement investment. This is clearly a difficult path. However, it is also clear that the actual level of CO_2_ emissions related to OPC production is not compatible with the neutrality goals set by the authorities for the cement sector. Therefore, the development of new types of binders as alternatives to OPC may play a role of paramount importance in the quest for cement sector carbon neutrality. Before deepening into some of the new binders commonly presented as alternatives to OPC, we should revisit the CaO-SiO_2_ system, which is the chemical base of OPC and the reason for its own success during the last century.

## 2. The Chemistry of the CaO-SiO_2_ System

**Figure 1:**
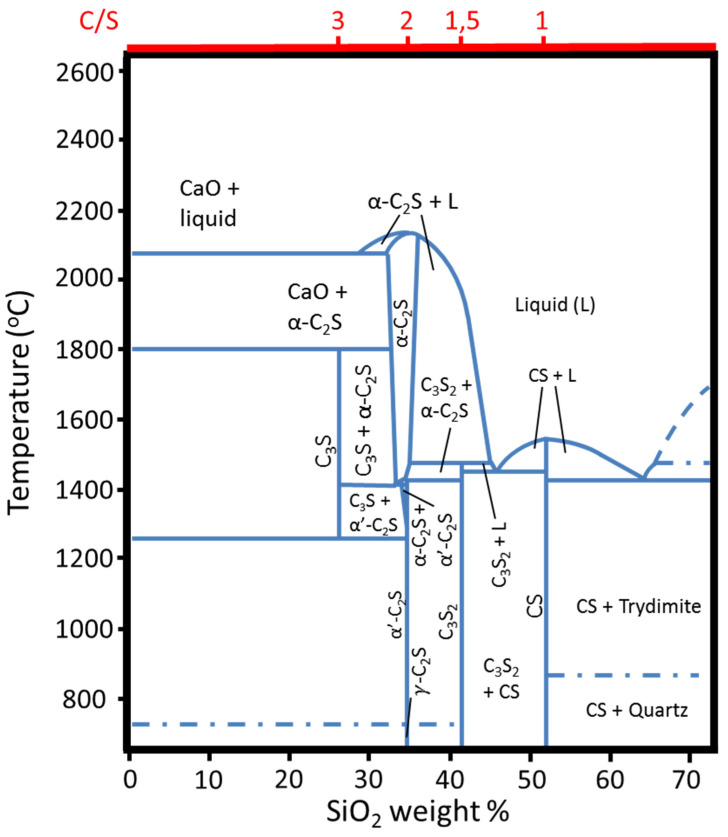
Detail of the CaO-SiO_2_ equilibrium phase diagram (adapted from [25]).

To frame the question of CO_2_ emissions in the production of hydraulic binders, it is important to shortly revisit the binary CaO-SiO_2_ system [25] (Figure 1), which is the chemical base of OPC and, to some extent, the ACTs that we will discuss further on.

There are two important characteristics of this system that impact the CO_2_ emissions related to the production of hydraulic binders, such as OPC.

The first one is that CaO does not abundantly exist in the earth’s crust and must be synthesized from limestone (CaCO_3_), according to the reaction:

(1) $${\mathrm{CaCO}}_{3}\to \mathrm{CaO}+{\mathrm{CO}}_{2}$$

Reaction (1), the decarbonation of limestone, is the cause behind the largest source of CO_2_ emissions in cement production. Thus, reducing the amount of CaO in the binder results directly in a reduction of CO_2_ emissions.

The second one is the hydraulic ability, or reactivity, of the phases that are present in the CaO-SiO_2_ system. Besides the main oxides, CaO and SiO_2_, in their different allotropic forms, there are four monophasic domains in this system: alite or (CaO)_3_SiO_2_, belite or (CaO)_2_SiO_2_, with five allotropic forms: α, α’_H_,α’_L_, β and γ, rankinite or (CaO)_3_(SiO_2_)_2_ and wollastonite or CaO·SiO_2_, which has two allotropic forms, α and β (from now on, we will use the common nomenclature used in cement literature; C_3_S for alite, C_2_S for belite, C_3_S_2_ for rankinite and CS for wollastonite. We will also use A, for Al_2_O_3_, F, for Fe_2_O_3_, Ŝ, for SO_3_ and finally H, for H_2_O).

**Figure 2:**
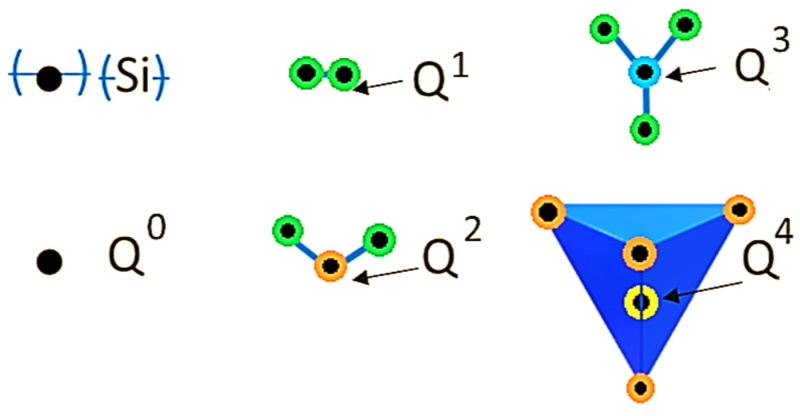
Coordination of different silica species in the CaO-SiO_2_ system.

OPC clinkers are formed essentially by alite, with smaller proportions of belite. However, from the binary diagram above, it is evident that producing clinkers in the rankinite or wollastonite domains would result in a decrease in CO_2_ emissions. It happens that only alite and belite are hydraulically active [26], with alite presenting higher reaction kinetics when compared to belite. That is, as the amount of SiO_2_ increases, the hydraulic reactivity of the calcium-silicate phases decreases. The reason for that is the fact that there are significant modifications in the structure of the calcium-silicate crystalline phases as the C/S ratio decreases, from 3 (alite) to 1 (wollastonite). These changes are essentially related to the organization of the SiO_x_ units in the crystals, as well as to the spatial distribution of the Ca atoms in the crystal network [27]. Whereas in alite and belite polymorphs, the [SiO_4_]^4−^ groups are isolated and present some degree of disorder in the superstructure, in rankinite, the Si and O atoms are arranged in sorosilicate groups [Si_2_O_7_]^6−^, in which a central O connects with two SiO_3_ units, whilst in wollastonite polymorphs, Si and O atoms are arranged in long parallel dreierkette-type chains of {[SiO3]^2−^}n units and there is an increase in the number of the bridging oxygen atoms in comparison to rankinite [27]. This structural shift among the four types of calcium silicate crystals (alite, belite, rankinite and wollastonite) can be summarized using the Q^n^ terminology, that designates how a given silica tetrahedron is connected to another silica tetrahedron in a given crystal, as shown in Figure 2: Q^0^ (black), Q^1^ (green), Q^2^ (orange), Q^3^ (blue) and Q^4^ (yellow), depending on how many O-Si species are connected to the central silicon atom. While in the hydraulically active phases, alite and belite, the coordination is Q^0^, in the non-hydraulically active phases, rankinite and wollastonite the coordination is Q^1^ and Q^2^, respectively. Experimental NMR results indicated that the Q^0^ units dissolve preferentially during the hydraulic reactions, resulting in C-S-H structures, predominantly formed Q^1^ and Q^2^ units [28].

Alite constitutes 50–70% of OPC and, upon hydration, is responsible for the strength development up to 28 days [29]. Belite may constitute 15–30% OPC and its contribution to the compressive strength can only be seen at later ages [29]. Both alite and belite, when hydrated, form a non-stoichiometric calcium silicate hydrate phase (C-S-H) and, as a secondary product, calcium hydroxide Ca(OH)_2_ or portlandite.

The C-S-H typically obtained from the hydration of alite or belite presents a C/S ratio of approximately 1.7 [30], with the excess of calcium, inevitably producing around 15–20% wt of portlandite [31]. However, the C/S ratio theoretically depends on the concentration of Si and Ca ions that are in the solution and can change from 0.8 to 2.1 [32]. Furthermore, through computational calculations, Pellenq et al. [33], reported that a binder with a C/S ratio between 1 and 1.1 would present crystalline domains characterized by improved mechanical performance.

The hydraulic reaction of OPC is an exothermic process that releases approximately 250 J/g of cement during the first 72 h of hydration [34]. This reaction starts with an induction period, characterized by an initial dissolution of species that are released into the aqueous solution. The species continue to dissolve until their concentration reaches a saturation condition, further leading to the nucleation and growth of the hydrated phases. At this point, there is an accelerating period in which occurs an extensive heat release, corresponding to the massive precipitation and growth of C-S-H and portlandite phases [35]. The precipitation and growth of these phases will result in the setting of the binder and in the increase of its mechanical strength with time. This is the basis of the alite/belite-based binders, the group to which OPC belongs.

The next section will briefly describe the binders that, although in different stages of development, have the potential to become alternatives to OPC in a perspective of CO_2_ emissions reduction.

## 3. ACTs: Alternative Clinker Technologies

The seek for carbon neutrality in the cement industry in the medium to long term will certainly depend on the possibility of scaling-up alternative clinker technologies (ACTs) encompassing new binder formulations and also new technological processing routes. The reason for that is the fact that hydraulic binders are a commodity whose demand will increase for sure in the next decades, namely with the economic development of large population countries in Asia, South-America and Africa [36]. Therefore, the cement industry cannot depend only on the availability of SCMs for large mass production of hydraulic binders or on the development of affordable CCS, it should pursue also the possibility of developing hydraulic binders with near to zero CO_2_ emissions. This long-term goal in the cement industry can only be obtained by reducing substantially the calcium content of the binder and by combining it with processing technologies that enable the full electrification of the processes. How far are we from this goal? In the next points, we will revisit the state-of-the-art in view of the possible answers to this question, having in mind that the main requirements for ACTs that can replace OPC as a widespread commodity material are:

- The capacity to mimic OPC properties, namely the production of mortar, concrete and reinforced concrete.
- A reduction of CO_2_ emissions large enough to justify the substitution of the BAT for cement production, since this represents a widespread technology with thousands of dedicated plants all over the world.
- A small impact on the need for modifications of national and international standards for cement use.

According to some authors [14], it will be difficult to turn around these requirements in order to develop a binder completely different from OPC in a short-term period, adapting it to environmental needs. In fact, to be able to achieve a significant breakthrough in this area, a large investment in research and large-scale demonstration projects is necessary. In addition, it will also be necessary to educate and train the consumers for the acceptance of these novel binders in the construction market [12]. However, even after facing all these difficulties for a long period of time, the hope of producing cement with near-zero CO_2_ emissions still remains, with a considerable number of ACTs continuously emerging. Thus, the investigation on this topic has significantly increased in the last two decades and, as a result, a number of alternative binding materials, different from OPC not only in formulation but also in their production routes and even application, have emerged and have been reported in the literature.

At the time this paper is written, it is difficult to say which of these ACTs, if any or all of them, can become an alternative to OPC in the near future, but certainly, the need for substantial CO_2_ reduction in cement production, together with the increasing demand for cement in the world, will bring this forward as a key question. The next paragraphs will be dedicated to a review of the alternative binder technologies that presently exist, with some of them being reported in the scientific literature as possible alternatives to common OPC [37]. This paper will be focused only on ACTs, without considering activated slags and geopolymers, since we believe that this is a sufficiently broad field to justify a focused review (e.g., for a recent deep review on this topic, see Provis et al. [38]).

### 3.1. Belite-Rich Clinkers

Belitic binders are not a recent discovery. In fact, they have been used since the times of the Roman Empire [39].This type of clinkers has essentially belite in its constitution and, therefore, its reaction process requires 10% less limestone, as it results from the analysis of Figure 1. Also, the synthesis of belitic clinkers requires lower processing temperatures, compared with the alitic-based clinkers, which also translates into a reduction of the CO_2_ emissions resulting from the furnace heating [39]. In addition, its lower heat of hydration [40], its better rheological properties and its improved durability at later ages, due not only to the smaller proportion of CH that is formed in the hydration but also because of its densely packed structure are advantages of belitic clinkers when compared to alitic ones.

Nevertheless, belite clinkers present a low early-age strength, due to their slower hydraulic kinetics. In fact, Kotsay et al. [39], reported, that after 28 days, the degree of hydration of the belite can be four times lower compared to the alite phase and, only after one year of hardening the strength of alite and belite hydrates are comparable. There are two main reasons for the lower hydraulic reactivity of belite at early ages: one is that the H_2_O molecules have more difficulty in penetrating the belite lattice, due to its densely packed structure [39], the other is that the Ca^2+^ ions attached to the SiO_4_ tetrahedron are less easily dissolved [41]. Therefore, the first step of the hydraulic reaction, the dissolution step, is slower in belite, as compared with alite. It has been disclosed that the incorporation of metal oxides into the lattice of belite, as substitutes of Si, increases the hydraulic reactivity at early ages of belite-rich clinker due to a higher infiltration of H_2_O molecules into the lattice, accelerating the dissolution of the material [42].

A completely different approach to belitic clinkers was proposed some years ago in which hydraulic binders with C/S = 1.4 were produced by inducing the formation of a dendritic belite phase embedded in an amorphous calcium-silicate phase [43]. These hydraulic binders were produced by a process involving heating the raw materials with a specified C/S ratio to a temperature below the liquid’s surface, followed by a two-step cooling ramp, in order to obtain during solidification a dendritic morphology of the crystalline phase. After milling the clinker obtained by this process, and by adding up to 25% of water, the paste set, showing mechanical performance that went up to four times higher than the values obtained for a reference round shape belite clinker, opening the possibility of developing a novel belite-based clinker with increased reactivity.

Therefore, belitic-rich clinkers can be used in conditions when factors such as low heat release and high later age strength are important parameters, for example in high-performance concrete, or large volume structures [40]. Nevertheless, although the substitution of alite by belite may reduce CO_2_ process-related emissions by up to 10%, it is still far from the goals defined for the cement industry in the global agenda for climate change.

### 3.2. Calcium Sulfoaluminate Cements and Belite-Ye’elimite-Ferrite Cements

Calcium sulfoaluminate cements (CSACs) are a belitic type of cement, which were developed in the 1970s [44] with the intention of compensating for the lower early-age strengths typically observed in belite-rich cements [44]. Typical raw materials used in the production of CSACs are limestone, calcium sulfate and aluminum-rich minerals or industrial by-products. Its production is carried out at temperatures around 1250 °C, approximately 200 °C lower than the necessary to produce OPC clinker [44], and is generally easier to grind [45].

The main clinker phases in CSACs are ye’elimite, Ca_4_(AlO_2_)_6_SO_4_, belite and calcium sulfate CaSO_4_ [44]. Since ye’elimite rapidly hydrates, it compensates for the loss in early-age strength in belitic clinkers [46]. As ye’elimite dissolves it enables the reaction with calcium sulfate and water and allows the formation of ettringite (Ca_6_Al_2_(SO_4_)_3_(OH)_12_·26H_2_O) and microcrystalline aluminum hydroxide Al(OH)_2_ [45]. Ye’elimite contains about 50% wt. of Al_2_O_3_ thus, the required alumina content in the raw materials to produce CSACs is above 20%, which can come from sources such as bauxite or industrial by-products, such as the ones proposed in the work by Canbek et al., namely red mud and sulfate-rich/high-lime fly ash [46]. However, the availability of low-cost sources of alumina-rich raw materials is certainly a limitation for the generalized use of CSACs [46].

Cement with high ye’elimite contents (>50% wt) can be used in combination with OPC to produce a fast-setting, rapid hardening cement [45]. CSACs cements with less ye’elimite (25–50% wt) contain significant amounts of belite (30–50% wt) and ferrite (5–20% wt) and can be a sustainable replacement material for OPC [45]. When compared to OPC, in some areas, the use of CSACs has been shown to have better performance when applied to concrete. They present lower shrinkage, lower cracking and higher resistance to freeze-thaw damage [45]. 

Another alternative to the CSACs is belite-ye’elimite-ferrite cement (BYFC), which presents a lower cost than CSACs, achieved by reducing the use of the most expensive aluminum-rich raw materials, resulting in a higher proportion of silicate and ferrite phases [47]. The ferrite phase, 4CaO·Al_2_O_3_·Fe_2_O_3_ has a slower hydration process than Ca_4_(AlO_2_)_6_SO_4_, therefore, ye’elimite, anhydrite and gypsum are the first phases to react, followed by ferrite and belite [48] Since the hydration of ye’elimite is faster than that observed for belite, the increase of compressive strength is similar to OPC [49].

Both CSACs and BYFCs can be produced in common clinker plants, essentially by changing the raw materials that are used to feed the kiln [47].This is a major advantage in terms of investment cost since it would allow the production of both types of material within the same facilities without the need for substantial process modifications. However, both CSACs and BYFCs present susceptibility to the carbonation process, caused by the dissolution of the atmospheric CO_2_ into the pore paste. This reacts with the hydrated products causing an increasing CO_3_^2−^ ion concentration. The formation of this anion has severe consequences facilitating the deterioration of ettringite [45], and raising the acidity of the system leading to the corrosion of steel rebar, used to reinforce concrete [44].

### 3.3. The Solidia Cement Approach

Solidia Cement patented in 2016 [50], is a non-hydraulic binder produced using the same raw materials as OPC, but with a lower amount of CaCO3 and a kiln temperature around 1200 °C, which allows a reduction of the CO_2_ emissions by 30% [50,51]. This binder has an overall C/S molar ratio of ~1 and it is formed essentially by wollastonite/pseudowollastonite, with smaller amounts of rankinite (13% wt), and belite (~3% wt) [52]. This mixture of calcium silicate phases has the ability to harden by a carbonation process and, consequently, there is no need for water consumption for the reaction to occur [51].

This cement is produced by feeding the granulated raw material into a natural gas-fired rotary kiln. The calcium silicate compositions created in the rotary kiln emerge in a “clinker” form, that is, in small granules with diameters of approximately 1 to 4 mm. The clinker is then ground to a powder with a mean particle size of approximately 12 μm. To produce concrete, this material is mixed with aggregates, sand and water. The cure of the concrete takes place when the mixture is exposed to a high-concentration gaseous CO_2_ environment (60–90%) [53] which allows the reaction of the binder phases and the production of CaCO_3_ and SiO_2_.

One of the most interesting characteristics of this binder is precisely the fact that the curing process can capture up to 300 kg of CO_2_, per ton of binder [51] and is only limited by the ability of gaseous CO_2_ to diffuse throughout the particles [51]. To speed up the curing process heat may be applied, these temperatures, if needed, can even be higher than 60 °C since there is no formation of ettringite [53]. The CaCO_3_ that is formed fills the pore space within the concrete, creating a dense microstructure and, the SiO_2_ is formed at the outer surface of the reacting cement particle [51].

Although Solidia Cement does not hydrate, water plays an important role in its forming and curing mechanism. Water contributes to the good flowability of the material and also acts as a permeating agent contributing to the cure development that occurs through a counter diffusion process where water molecules are replaced by CO_2_ molecules [53]. However, since the water is not consumed, 90% of it can be recovered, while the remaining is retained in the cured concrete [52]. The mechanical properties of the concrete are equivalent to those of OPC, and they are achieved within a shorter curing period [52]. Another characteristic of this binder is that the carbonation process only releases about 87 kJ/mol of heat during curing which is dissipated through the evaporation process of the water that is used in the concrete preparation [53].

Even though this is a promising cement, its application and use are limited, since its curing process must be conducted under very controlled CO_2_ concentration conditions, which, so far, can only be provided in a ready-mixed concrete plant [12] impairing to some extent the generalized use of Solidia cement as a substitute of OPC.

### 3.4. The Celitement Approach

Celitement^®^ is a patented hydraulic binder, developed by the Karlsruhe Institute of Technology (KIT) in collaboration with the SCHWENK Zement KG industry [54]. Its concept is to synthesize and stabilize a short-time precursor of C-S-H to produce a hydraulic binder [55]. This material is characterized by its low energy demand during its production process, which enables a reduction in CO_2_ emissions [55]. The Celitement production relies on the formation of an intermediate phase that is prior to the development of the C-S-H. This intermediate phase has a similar structure to C-S-H, but a slightly different chemical composition and is referred to as hydraulic Calcium Hydro Silicate (hCHS) [56].

The production method of Celitement, uses raw materials CaO, in the simplest case, or Ca(OH)_2_ and quartz sand [57]. It requires a calcination stage (around 1000 °C) that is applied only to the CaCO_3_-rich raw-material, and hydrothermal processing of the raw mix that takes place in an autoclave at a temperature of 200 °C and at a saturated steam pressure of 12 bar, which facilitates the full electrification of the process. The product that results from the autoclave is stabilized by a strong hydrogen bond which makes it non-hydraulically active [55]. In a second step, this product goes through a special grinding operation [55] that enables the destruction of the hydrogen bonds [57] and, around the cores of the non-reactive co-milled silicates, a new amorphous calcium hydrosilicate (hCHS) is produced [55,56,57]. The final produced material is mainly amorphous, with highly disordered phases and high specific surface, containing in its composition Q^0^ and Q^1^ silicate species [57,58]. After 17–20 h of hCHS hydration, a well-ordered C-S-H phase is formed, releasing a very low heat of hydration (120–150 J/g) and promoting an early-age strength comparable to OPC [55].

The Celitement approach is based on a technology that completely differs from the one known today for the production of Portland cement, leading to what may be a significant drawback in its industrial implementation. Nevertheless, this new technology is already under the demonstration phase, with a recent expansion to the pilot plant, constructed in 2011, allowing the production of approximately 700 kg per day [59].

### 3.5. The C/S≈1 Amorphous Approach (X-Clinker)

Another alternative, developed and patented internationally by CIMPOR and Técnico-Lisbon, is an amorphous low-calcium hydraulic binder characterized by a raw mix containing 33% less CaCO_3_ than the typical OPC, and an overall C/S ratio of 1, allowing for a reduction of more than 25% of the usual OPC clinker process-related CO_2_ emissions [60,61].

The production process of this binder allows the use of traditional raw materials, such as limestone, clay, marl, sand, etc., and consists in fully melting the raw mixture, at a temperature of 1550 °C, followed by a rapid cooling [59]. The resultant product is mostly amorphous (~94% wt), with the presence of a small amount (<10%) of pseudo-wollastonite [60]. It should be pointed out that the full melting of the mixture may facilitate the electrification of the process through plasma or electrical arc melting, which may lead to a scenario where the effluent gas stream is solely fed by the CO_2_ generated by the calcination of the raw meal. Such a highly CO_2_ concentrated gas stream could potentially be combined with green H_2_ to produce methanol and other hydrocarbons [62,63].

The reactivity of this novel binder comes, mostly, from its amorphous phase, yet even though existing in a small amount, the presence of pseudo-wollastonite has been shown to have some influence on the hydrated product performance [64]. Better compressive strength results were obtained when this phase was produced in an amount of ~6% wt [64]. An investigation on the hydration of this binder observed, by ^29^SiMAS-NMR spectroscopy, that the least coordinated Q^n^ units, Q^0^ and Q^1^, play a very important role in the hydration since they appear to be very prone to polymerize and convert into C-S-H structures that are similar to tobermorite [60]. By changing the structure from crystalline to amorphous, the arrangements of Si–O bonds become more disordered, which favors their dissolution [60] and, consequently, the further precipitation of equilibrium hydration products [65].

The behavior of this novel amorphous binder was further studied by Santos et al. [28], which investigated the effect of different alkaline activators (Na_2_CO_3_ and a mixture of NaOH and Na_2_SiO_3_) on the mechanical strength and structural characteristics of hydrated pastes. It was observed that, when activated, those pastes presented increased hydration kinetics, allowing for an improvement in their mechanical performances. Furthermore, the most competitive results were obtained when the pastes were activated with Na_2_SiO_3_, with a 3 wt% total content of Na_2_O, obtaining pastes with strengths comparable with those of traditional OPC [28].

In terms of technological development, this approach presents a main drawback, which is the need for a pyro-processing step that is 100 °C superior to that of OPC and, the requirement of a sodium silicate solution for activation, in order to present competitive early-age strength.

In addition, since the processing conditions require the formation of 100% liquid phase, some adaptations to the usual BAT of clinker production may be required in order to industrially implement this type of technology.

### 3.6. Summary of the Alternative Clinker Technologies

**Figure 3:**
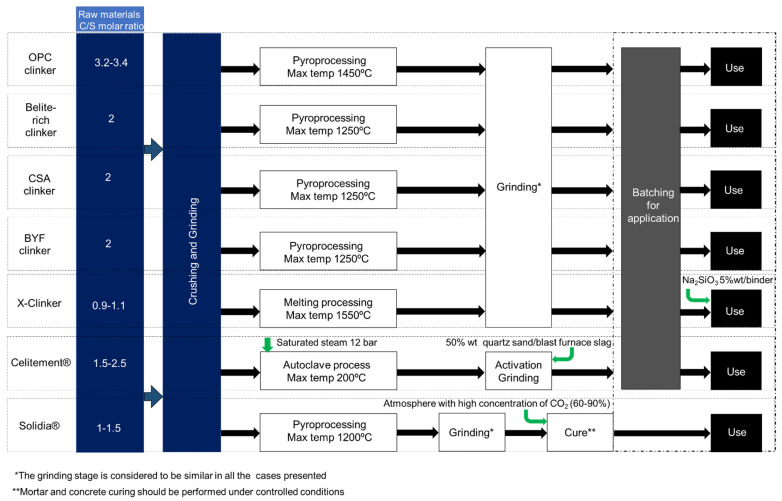
Simplified schematic representation of the stages considered within the production processes of the alternative binders reviewed.

Figure 3 shows a simplified flow chart containing the stages considered for the production of the alternative binders mentioned in the present review, evidencing the differences of the process in the various ACTs approaches. From Figure 3, it becomes clear that in the present state, research should be pursued in all the presented solutions. From the several technological proposals contained in Figure 3, it should be noted that only the Celitement^®^ and the X-Clinker approaches presently consider a scenario of full process electrification, while the other approaches essentially follow a fuel combustion-based design, similar to the existing BAT. The proximity to the BAT for clinker production is certainly an advantage for the industrial implementation of some of the alternative binders mentioned above, however, when looking forward through a perspective of cement industry decarbonization, it is the author’s belief that full or partial conversion of the existing fuel-based technology to electricity-based technology should occur in the next decades.

**Figure 4:**
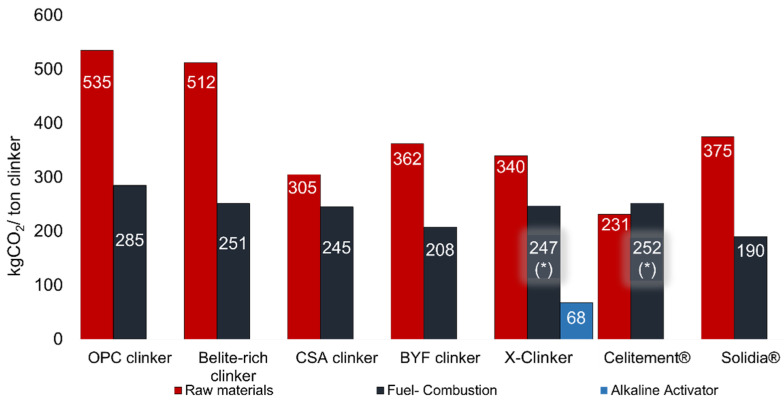
CO_2_ contributions for the energy- and material-related emissions of the various alternative binders reviewed. NOTE: (*) indicates the technologies that already consider a fully electrified production process and therefore, depending on the nature of the electricity used, the thermal component of CO_2_ emissions can be completely eliminated.

Figure 4 shows a detailed description of the various sources of CO_2_ emissions within the production processes considered for the ACTs reviewed, evidencing the separation of the contributions for the thermal and material-related emissions. The CSA, X-Clinker and Celitement approaches lead to material-related CO_2_ emissions smaller than 0.35 tons per ton of clinker. If full electrification of the process for these ACTs with green electricity is achieved, a target for CO_2_ emission in cement production smaller than 0.25 tons of CO_2_ per ton of cement is within reach of the cement industry in the upcoming years.

## 4. Electrification of the Cement Production Process

Certainly, the reduction in the ecological footprint in the cement industry encompasses the substitution of fossil fuel in the cement kiln for electrification in the heating process. In fact, as the cost of solar photovoltaics, wind power and battery storage decreases, the coupling of industry electrification with renewable electricity supply has become a potential solution for industrial decarbonization [66]. In particular, the electrification of the cement production has become a more feasible alternative to the current method of combustion, and unlike gas furnaces, electric furnaces have very low direct emissions of CO_2_, NOx and SOx, however electric furnaces may have shorter lifetime periods than conventional furnaces [67]. According to the work of Madeddu et al. [68], the cement industry has the potential for 36% electrification, which essentially considers the calcination of limestone, whilst the energy necessary for clinker burning is not considered electrifiable at this stage of technology development. However, in the 2018 report of the CemZero project [69], it is referred that the production of cement clinker by means of plasma technology appears to be technically possible. This CemZero project is being conducted in Sweden, as a collaboration between Cementa and Vattenfall, and aims to test different technologies for the total or partial electrification of the cement production process.

Their study indicated that the production costs of cement in an electrified process appear to be doubled in comparison to today’s technology but could be competitive compared to other technological options for radical emission reductions [70].

Another project that points to an electrically powered calcination process is the LEILAC, which is being developed by a large consortium of companies and foresees to implement a fully operational plant by 2023 [71]. This project assesses, through a direct separation perspective, the feasibility of CO_2_ capture and storage, by means of the generation of a highly CO_2_ concentrated gas stream from the calcination stage important development is the electrochemical approach that converts CaCO_3_ in Ca(OH)_2_ and may represent a real alternative solution to overcome the CO_2_ emissions from the decarbonation process in cement industry. This technology is able to capture excess power and, through electrolysis, convert limestone into hydrated lime, working similarly to a battery. This process was already tested at the laboratory scale, and they reported that a future implementation on a larger scale could allow for a decentralized, automated, easy to deploy, easy to start-up and shut down cement plant [72]. Processes of combining calcination and CO_2_ capture by using electricity were also reviewed recently by Tokheim et al. in a conference paper [73]. Due to the critical importance of the topic of electrification in the framework of ACTs a short review of these processes is presented in the following points.

### 4.1. Plasma Technology

This technology can produce temperatures above 2000 °C and is currently used as waste treatment and in some niches in the steel industry [74]. Its implementation in the cement industry may open the possibility of using recycled CO_2_ as plasma gas [75], which further benefits the goal of CO_2_-free cement industry. The main disadvantage of using thermal plasma is the overheating of the reaction media [76], which can impair the clinker performance by changing the phases present at room temperature, and also the short lifetime of the electrodes. As referred above, the use of plasma was one of the technologies that CemZero tested on a laboratory scale, being reported that it is possible to produce cement clinker of regular quality by using plasma gas as a heat source. Nevertheless, a larger-scale test must be performed, since one concern with the use of this technology is how to maintain the rate of heat transfer in a rotary kiln [69].

### 4.2. Resistive Electrical Heating

In this approach, a metal surface is heated by running a current through a resistive element, which usually is protected by a shroud. Then, the heat can be transferred either by gas, through high-velocity convection, or directly to the material, through radiation or conduction, if it is possible to promote the direct contact between the raw meal and the resistive heater. This type of technology is already used in glass melting furnaces and can either be applied in combination with another heating method, acting as a boost in gas-fired furnaces [77], or as a complete replacement of the traditional heating method, providing a completely electrified production [78,79]. Typically, electric glass furnaces use a vertical melting process [80]. The furnace is continuously fed at the top side and the melted material leaves the furnace through the bottom. The heating provided by running a high current into molybdenum electrodes [80] whose configuration arrangement influences the homogenization capacity of the mixture [81]. Jebava et al. [81] reported that by distributing the electrode into a longitudinal central row, there is an optimization on the utilization of space and the highest melting performance. These types of furnaces can provide an efficiency of up to 87% and a wide range of high-temperature processes [82].

### 4.3. Electromagnetic Heating

By using electromagnetic waves, the electromagnetic heating technologies are able to provide high temperatures, with an efficiency of up to 90% [82]. Furthermore, it has the advantage of being able to generate rapid heat within a target material [82]. Some examples of these technologies include:

#### 4.3.1. Induction Heating

Induction heating occurs when an electrically conducting object is placed in a varying magnetic field. The friction between molecules when the material is magnetized first in one direction, and then in the other is converted into heat [83]. To cool the induction furnace, water-cooled coils are used. This technology allows almost instantaneous heating or cooling of the calciner. However, there is a risk of overheating the product when the heat necessary for the calcination is inferior to the heat provided by the induction system [84]. This type of technology is able to reach high temperatures fast and is currently applied in induction furnaces used for metal melting [85]. However, it is not currently seen as a potential solution for processing ceramic materials [82].

#### 4.3.2. Microwave Heating

The heat necessary for calcination can be delivered by microwave through a radiation form, by transferring direct energy in the form of electromagnetic waves into the material. Corrêa et al. [86], investigated the use of a microwave oven in the calcination process reaching a temperature of 1160 °C. The team reported that this method provides better results in terms of reaction time, energy consumption and emission of polluting gases when compared with traditional methods. Furthermore, the use of a refractory ceramic coated with copper oxide reduced the energy expenditure and accelerated the process to be twice as fast. Microwave heating is currently used in the conversion of biomass and by-products and also, in waste processing, although mostly at the laboratory scale [87]. Even though this technology is able to provide a rapid internal heating of large volumes, large production scales presents high operation costs, which presently hinder its use in a generalized form [82]. 

### 4.4. Benefits and Difficulties of Electrification

It has to be taken into consideration that the electrification of any industry is highly influenced by energy and environmental policies [74]. Not only, can the electrification cost be challenging, but also the electricity prices must be competitive. Moreover, industry electrification only reduces greenhouse gas emissions if renewable-generation capacity is added to meet its electricity demand. Nevertheless, in the current conditions of climate change, a complete shift from an oil and gas economy to a green energy source has been associated with the future of energy [88]. In fact, the global renewable energy generation capacity has progressively been increasing, and it is estimated that by 2050, more than 80% of electricity will come from renewable sources [89]. Currently, in the EU-27, renewable sources already make 34% of electricity consumption, with the majority coming from wind and hydropower and a small part from solid biofuels and solar power [90]. Hence, as the prices of renewable electricity and electric equipment continue to drop [66,91], the electrification of the cement industry can be an important option to achieve a high reduction of CO_2_ emissions.

## 5. Final Remarks

To be able to seek neutrality in the cement industry, the current method of cement production must change. There are many potential routes to lower CO_2_ emissions, and some are more prone to succeed than others. This paper reviewed and discussed a variety of alternative clinker technologies for the partial or complete substitution of OPC. The performance of these materials is largely dependent on their physical and chemical characteristics, which have a major influence on its curing process and, consequently, on the binder’s mechanical performance. In some of the cases, further studies for a complete characterization of materials properties are needed.

Nevertheless, the goal of CO_2_ neutrality in the cement industry can only be reached through an extended replacement of OPC by green alternative binders, as well as through a transition from fossil fuel technology to a green energy-based technology.

The CO_2_ process-related emissions, assuming that the main calcium source is limestone, are shown for different ACTs, and very significant differences among these materials can be observed. It should be noted that, if one considers that limestone is the source of Ca in the clinker, this is an absolute limit. As it can be observed, the production of alite or belite-based clinkers will always result in CO_2_ emissions above 500 kg/ton of clinker, while the CSA, BYF and Solidia^®^ cements allow a reduction of the CO_2_ process-related emissions below 400 kg/ton, keeping the same clinker production technology. Still, below the 400 kgCO_2_/ton of clinker, it is possible to find the alternatives X-Clinker and Celitement^®^, with both technologies being based on fully electrified processes. Concerning “wollastonitic” clinkers, two radically different approaches exist: the Solidia Cement approach, in which the setting of near-wollastonite phase specimens is achieved via carbonation, and the X-Clinker approach, in which instead of a crystalline material, the reactivity with water is achieved through the amorphization of the material with a composition close to wollastonite.

The direct emission of CO_2_ arising from materials decarbonation is an important factor but not the only one. In fact, the CO_2_ emissions arising from the firing process also play an important role in clinker production, which in the case of alite-, belite- and aluminate-based clinkers represent about 40% of the total CO_2_ emissions. In this way, the possibility of process electrification is also a key factor in what concerns the reduction of CO_2_ emissions in clinker production since this CO_2_ contribution could drop to zero if non-fossil sources are used for electric energy production. Under this scenario, the lower limit for the total CO_2_ emission would be defined by the own chemistry of each binder, as a result of the calcination of its raw materials.

Table 1 presents a summary of the potential impact of each ACT regarding energy- and process-related CO_2_ emissions, as well as some important characteristics of the alternative technologies and binders. In this table, it was considered that the Celitement^®^ clinkers are prone to electrification since they do not need a rotary kiln or considerably high temperature, which facilitates the implementation of resistive electrical or microwave heating. Likewise, the X-Clinker procedure does not implement a rotary and, even though there is the need for high temperatures, there is also a complete melting, which makes the overheating of the reaction media unproblematic in the production of clinker. Therefore, processes such as plasma heating or electrical arc are viable. All the other clinkers need a controlled high temperature and a rotary kiln, which makes the implementation of an electrification process challenging.

## Figures and Tables

**Table 1:** Technology-related and binder characteristics of the ACTs reviewed.

	Technology-Related Characteristics					Binder Characteristics		
Clinker	Process-Related CO_2_ (kg/ton)	Energy-Related CO_2_ (kg/ton)	Total CO_2_ (kg/tonne)	High Abundance of Raw Materials	Electrification Feasibility	Heat of Hydration (J/g)	Early and Late Age Strength	Curing Can Be Performed in Non-Controlled Conditions
OPC clinker	535 [92]	270 [92]	805	Yes	Challenging	250–350 [34]	Both competitive	Yes
Belite-rich clinker	512 [14]	251 [47,92]	763	Yes	Challenging	175–250 [93]	Long-time strength is competitive [39]	Yes
Calcium sulfoaluminate (CSA) clinker	305 [92]	245 [92]	550	No	Challenging	130 [94]	Both competitive [45]	Yes
Belite-ye’elimite-ferrite (BYF) clinker	362 [14]	208 [95]	570	No	Challenging	523 [96]	Competitive at early ages but not at long ages [97]	Yes
Celitement^®^ clinker	231 [56]	252 [56]	May go from 231 to 483	Yes *	Accessible	120–150 [55]	Both competitive [55]	No
Solidia^®^ clinker (crystalline CS)	375 [52]	190 [52]	565	Yes	Challenging	150 [53]	Both competitive [52]	No
X-Clinker (amorphous CS)	340 [98]	247 [92,98]	May go from 377 to 624	Yes	Accessible	125 [28]	Both competitive ** [28]	Yes **

* Even though the raw material of Celitement^®^ are lime and quartz, their use was conducted under laboratory conditions, with high purity raw materials [56,57]. ** Tests performed on pastes showed that the strength of the X-Clinker, compared with OPC, is competitive at all ages [27]. However, tests performed on mortars showed that, for the X-Clinker to be competitive at early ages (2 days), a cure at temperatures around 35 °C may be required [99].
